# Case Report: Acute biliary pancreatitis after semaglutide dose escalation in an adolescent with obesity and cholelithiasis

**DOI:** 10.3389/fped.2026.1959617

**Published:** 2026-09-07

**Authors:** Abdalrhman H. Alanizi, Ghadeer M. Alruwythi, Norah H. Alotaibi, Noof H. Alanazi, Amirah M. Alghurabi, Reem Qubaiban, Nawal Alsubaie

**Affiliations:** 1 Pharmaceutical Care Services, King Abdullah Bin Abdulaziz University Hospital, Riyadh, Saudi Arabia; 2 College of Pharmacy, Princess Nourah Bint Abdulrahman University, Riyadh, Saudi Arabia; 3 College of Medicine, Princess Nourah Bint Abdulrahman University, Riyadh, Saudi Arabia; 4 Department of Internal Medicine, King Abdullah Bin Abdulaziz University Hospital, Riyadh, Saudi Arabia; 5 Department of Saudi Health Counsel, National Cancer Center, Riyadh, Saudi Arabia

**Keywords:** acute pancreatitis, adolescent, biliary pancreatitis, cholelithiasis, GLP-1 receptor agonist, obesity, semaglutide

## Abstract

**Background:**

Semaglutide is increasingly used for weight management in adolescents with obesity. Although gastrointestinal adverse effects are common, gallbladder disease and acute pancreatitis are less frequent but clinically important complications. The relationship between semaglutide therapy, biliary disease, and pancreatitis in adolescents remains incompletely characterized.

**Case description:**

A 16-year-old female with obesity was treated with glucagon-like peptide-1 receptor agonists for weight management. Following approximately nine months of liraglutide therapy, she was transitioned to semaglutide and subsequently escalated to 2.4 mg once weekly, with substantial weight loss. An abdominal ultrasound performed before escalation to 2.4 mg demonstrated multiple small gallstones without biliary ductal dilatation. Approximately five weeks after dose escalation, she presented with severe epigastric pain, nausea, and vomiting. Laboratory investigations demonstrated elevated lipase, bilirubin, AST, and ALT. Ultrasound and magnetic resonance cholangiopancreatography confirmed multiple gallstones without choledocholithiasis or biliary ductal dilatation. She was diagnosed with acute biliary pancreatitis and managed conservatively. Semaglutide was discontinued, followed by laparoscopic cholecystectomy. Histopathology demonstrated chronic calculous cholecystitis. The Naranjo Adverse Drug Reaction Probability Scale yielded a score of 3, corresponding to a possible adverse drug reaction.

**Conclusion:**

This case highlights the clinical complexity of acute biliary pancreatitis occurring during semaglutide therapy in an adolescent with documented cholelithiasis. Although a definitive causal relationship cannot be established, the temporal relationship dose escalation and substantial weight loss raises the possibility that semaglutide may have contributed to the development of biliary complications and subsequent pancreatitis. Clinicians should remain vigilant for gallbladder disease and pancreatitis in adolescents receiving glucagon-like peptide-1 receptor agonists, particularly those with known gallstones or substantial weight loss.

## Introduction

Semaglutide is a long-acting glucagon-like peptide-1 (GLP-1) receptor agonist that promotes glucose-dependent insulin secretion, suppresses glucagon secretion, delays gastric emptying, and increases satiety. These effects contribute to improved glycemic control and weight reduction. Semaglutide is approved for chronic weight management in adults and adolescents with obesity and has demonstrated substantial efficacy in adolescents with obesity when administered at a maintenance dose of 2.4 mg once weekly (1, 2).

Gastrointestinal adverse effects, including nausea, vomiting, diarrhea, constipation, and abdominal discomfort, are commonly reported with semaglutide. Less frequent but clinically important adverse events include gallbladder disease and acute pancreatitis. The prescribing information for semaglutide identifies both acute pancreatitis and acute gallbladder disease as clinically important adverse events and notes that substantial or rapid weight loss may increase the risk of cholelithiasis (3).

Glucagon-like peptide-1 receptor agonists have been associated with an increased risk of gallbladder and biliary diseases in randomized clinical trials, with greater risk observed in weight-loss trials, at higher doses, and with longer treatment duration (4). With increasing use of glucagon-like peptide-1 receptor agonists for obesity management in adolescents, recognition of uncommon but potentially serious biliary complications in this population is increasingly important.

We report the case of a 16-year-old adolescent with documented cholelithiasis who developed acute biliary pancreatitis approximately five weeks after escalation of semaglutide to 2.4 mg once weekly. Although cholelithiasis provides a clear alternative explanation for the pancreatitis, the temporal relationship with semaglutide dose escalation and substantial weight loss during glucagon-like peptide-1 receptor agonist therapy raises the possibility that semaglutide may have contributed to the development of biliary complications and subsequent pancreatitis. This case highlights the clinical considerations surrounding biliary complications in adolescents receiving semaglutide.

## Case presentation

A 16-year-old female with a history of bronchial asthma and no previous surgical history was evaluated for obesity requiring pharmacologic weight management. She had no history of alcohol consumption. Liraglutide (Saxenda) was initiated at 0.6 mg once daily for one week and subsequently titrated to 3 mg once daily. She continued liraglutide for approximately nine months and experienced substantial weight reduction from 120 kg (BMI 46 kg/m^2^) to 97 kg (BMI 37.5 kg/m^2^).

During liraglutide therapy, she reported hair loss, intermittent constipation, and recurrent epigastric pain radiating to the back; however, no emergency department visits were documented during this period. Due to poor tolerance, liraglutide was discontinued and semaglutide was initiated for continued weight management.

Given her previous exposure to liraglutide, semaglutide was initiated at 1 mg once weekly rather than the conventional starting dose. The dose was subsequently increased to 1.7 mg once weekly for four weeks and then escalated to 2.4 mg once weekly. Her weight decreased from 97 kg at the time of switching to semaglutide to 93 kg during dose escalation and subsequently to 86 kg (BMI 32 kg/m^2^) at presentation.

An abdominal ultrasound performed on 30 October 2025, before escalation to semaglutide 2.4 mg weekly, demonstrated multiple small gallstones measuring up to 0.3 cm, without gallbladder wall thickening or biliary ductal dilatation (common bile duct, 0.4 cm). This finding established the presence of cholelithiasis before the subsequent episode of pancreatitis.

Approximately five weeks after escalation to semaglutide 2.4 mg weekly, she developed severe epigastric pain. On 9 February 2026, she presented to the emergency department with severe abdominal pain of one-day duration following ingestion of a fatty meal. She was alert, oriented, and hemodynamically stable. Examination revealed right upper quadrant tenderness and tenderness over Murphy's area. Initial laboratory investigations were unremarkable. She received intravenous morphine 3 mg and was discharged.

On 10 February 2026, she returned to the emergency department because of persistent and worsening abdominal pain despite the initial treatment. She reported nausea and two episodes of vomiting. Examination revealed epigastric tenderness with a negative Murphy's sign. She leaned forward because the pain worsened in the supine position. Vital signs remained stable (temperature, 36.7 °C; respiratory rate, 20 breaths/min; blood pressure, 107/72 mmHg; oxygen saturation, 100% on room air).

Laboratory investigations demonstrated elevated lipase (216 U/L), total bilirubin [46.9 µmol/L(2.74 mg/dL)], direct bilirubin [29.1 µmol/L(1.70 mg/dL)], AST (120 U/L), and ALT (127 U/L), supporting a diagnosis of acute pancreatitis with biliary involvement (Table 1).

**Table 1:** Serial laboratory findings during the clinical course of acute biliary pancreatitis.

Date	25/02/2025	09/02/2026	10/02/2026	11/02/2026	12/02/2026	13/02/2026	14/02/2026	15/02/2026
Laboratory parameter								
ALT (U/L); 0 - 35	24	7	127	114	80	67	70	66
AST (U/L); 0 - 35	13	12	120	69	29	29	46	40
Total bilirubin, µmol/L(mg/dL); 5-21 umol/l (0.29–1.23 mg/dL)	5.0 (0.29)	8.1 (0.47)	46.9 (2.74)	15.7 (0.92)	10.2 (0.60)	8.2 (0.48)	8.6 (0.50)	8.8 (0.51)
Direct bilirubin, µmol/L(mg/dL); 0–3.4 µmol/L (0–0.20 mg/dL)	0.9 (0.05)	2.1 (0.12)	29.1 (1.70)	6.7 (0.39)	4.0 (0.23)	3.3 (0.19)	2.7 (0.16)	2.6 (0.15)
Amylase (U/L); 22 - 80	32	—	—	—	—	—	—	—
Lipase (U/L); 11 - 82	23	—	216	31	22	—	—	—

Abdominal ultrasound demonstrated multiple small mobile gallstones, with the largest measuring 0.5 cm. The gallbladder wall measured 0.2 cm, with no pericholecystic fluid. The common bile duct measured 0.5 cm, and no biliary ductal dilatation was identified. The visualized pancreas appeared grossly unremarkable.

Magnetic resonance cholangiopancreatography (MRCP) confirmed multiple gallbladder stones without evidence of choledocholithiasis or biliary ductal dilatation. Mild diffuse hepatic steatosis was also noted, with an otherwise unremarkable pancreas.

The patient was admitted under the care of the general surgery team and diagnosed with acute biliary pancreatitis secondary to cholelithiasis. She was managed conservatively with intravenous Ringer's lactate, analgesia, antiemetics, Omeprazole, and prophylactic heparin. Semaglutide was held. Serial laboratory monitoring demonstrated gradual improvement, and she remained clinically stable throughout her admission. She was initially kept nil per os with maintenance intravenous fluids (5% dextrose in 0.45% sodium chloride), and her abdominal pain improved substantially by 13 February 2026.

On 15 February 2026, she underwent successful laparoscopic cholecystectomy and was discharged in good condition on postoperative day one. At outpatient follow-up, she was clinically well, with no nausea, vomiting, or fever. She was tolerating oral intake and passing bowel motions normally. Examination showed a soft, non-tender abdomen and a well-healed surgical wound. Histopathological examination demonstrated chronic calculous cholecystitis.

**Figure 1:**
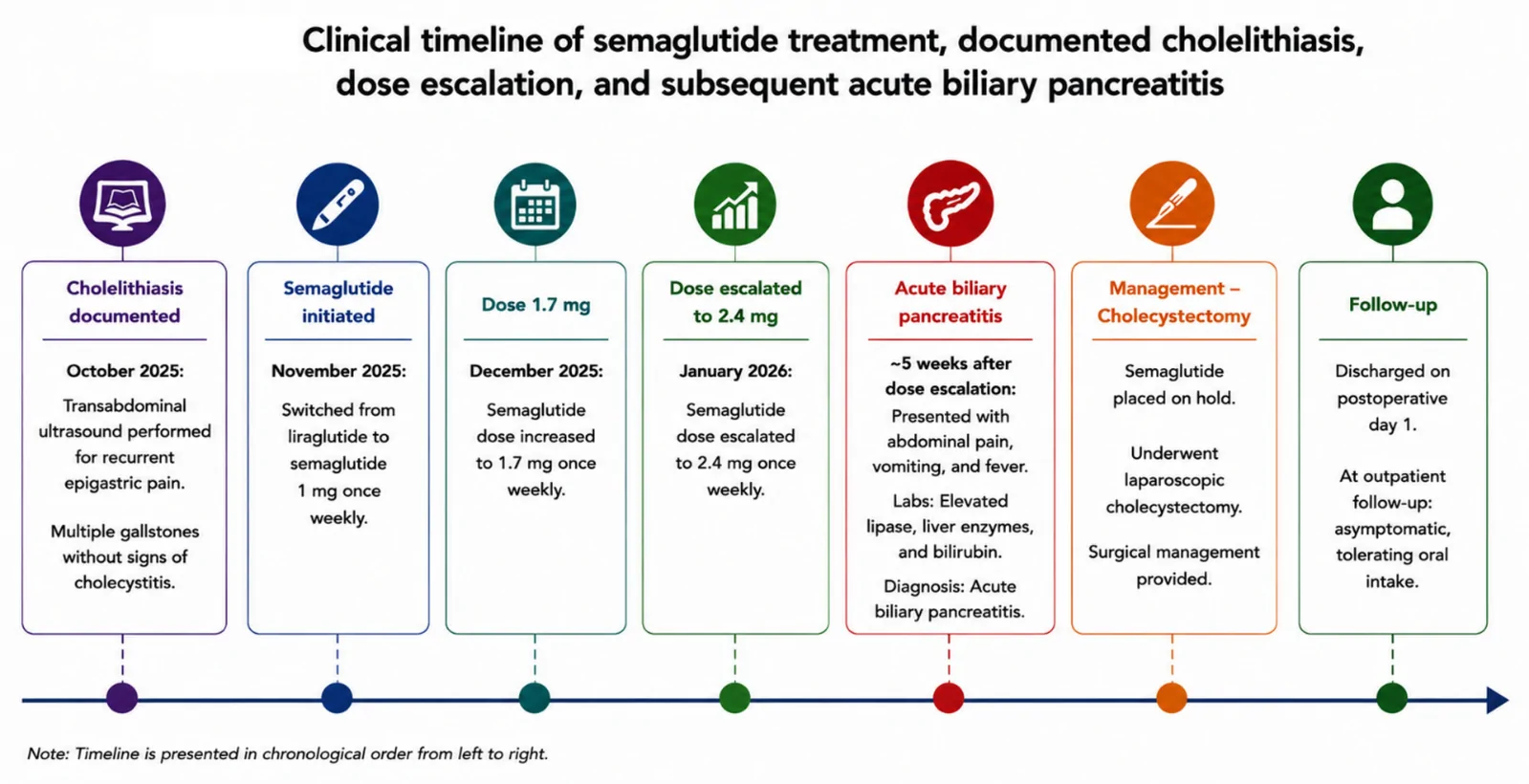
Clinical timeline of semaglutide treatment, documented choletihiasis, dose escalation, and subsequent acute biliary pancreatitis.

The key clinical events, including semaglutide initiation and dose escalation, documentation of cholelithiasis, development of acute biliary pancreatitis, and subsequent management, are summarized in Figure 1.

The Naranjo Adverse Drug Reaction Probability Scale was applied to assess the potential causal relationship between semaglutide and acute biliary pancreatitis. The patient had a total score of 3, corresponding to a possible adverse drug reaction (5). The individual components of the Naranjo assessment and the corresponding scores are presented in Table 2.

**Table 2:** Naranjo adverse drug reaction probability scale assessment for semaglutide and acute biliary pancreatitis.

No.	Naranjo question	Yes	No	Do not know	Score in this case
1	Are there previous conclusive reports on this reaction?	+1	0	0	+1
2	Did the adverse event appear after the suspected drug was administered?	+2	−1	0	+2
3	Did the adverse reaction improve when the drug was discontinued or a specific antagonist was administered?	+1	0	0	0
4	Did the adverse reaction reappear when the drug was readministered?	+2	−1	0	0
5	Are there alternative causes that could have caused the reaction?	−1	+2	0	−1
6	Did the reaction reappear when a placebo was given?	−1	+1	0	0
7	Was the drug detected in blood or other fluids in concentrations known to be toxic?	+1	0	0	0
8	Was the reaction more severe when the dose was increased or less severe when the dose was decreased?	+1	0	0	0
9	Did the patient have a similar reaction to the same or similar drugs in any previous exposure?	+1	0	0	0
10	Was the adverse event confirmed by objective evidence?	+1	0	0	+1
	Total				3

## Discussion

Semaglutide is a glucagon-like peptide-1 receptor agonist with established efficacy for weight management in adolescents with obesity. In the pivotal randomized controlled trial involving adolescents aged 12 to less than 18 years, once-weekly semaglutide 2.4 mg resulted in substantial reductions in BMI and body weight compared with placebo (1). Liraglutide, another glucagon-like peptide-1 receptor agonist, has also demonstrated efficacy for weight management in adolescents with obesity (2).

Despite their therapeutic benefits, glucagon-like peptide-1 receptor agonists have been associated with gallbladder and biliary complications. A systematic review and meta-analysis of 76 randomized clinical trials involving more than 100,000 participants found that glucagon-like peptide-1 receptor agonist therapy was associated with increased risks of gallbladder or biliary disease, cholelithiasis, and cholecystitis. The association was more pronounced in trials evaluating weight loss, higher doses, and longer treatment duration (4). These findings are particularly relevant to the present case because the patient experienced substantial weight loss during treatment and developed a biliary complication following escalation to the highest recommended weekly dose of semaglutide.

The temporal relationship between semaglutide dose escalation and the onset of acute pancreatitis raises the possibility of a contributory association. However, gallstones were documented before the episode of pancreatitis and represent an important alternative explanation for the event. The occurrence of acute biliary pancreatitis approximately five weeks after escalation to semaglutide 2.4 mg weekly, together with substantial weight loss during glucagon-like peptide-1 receptor agonist therapy, raises the possibility that semaglutide may have contributed to the development of biliary complications and subsequent pancreatitis. This potential association is biologically plausible given the association between glucagon-like peptide-1 receptor agonists and gallbladder disease and the recognized risk of gallstone formation during substantial or rapid weight loss (3, 4).

The Naranjo Adverse Drug Reaction Probability Scale yielded a score of 3, corresponding to a possible adverse drug reaction (5). The assessment was limited by the presence of an alternative cause—documented cholelithiasis—and by the inability to establish improvement specifically attributable to semaglutide discontinuation because the patient subsequently underwent definitive treatment with cholecystectomy. Therefore, although a contributory role of semaglutide cannot be excluded, a definitive causal relationship between semaglutide and pancreatitis cannot be established.

Evidence specifically addressing pancreatitis in adolescents receiving semaglutide remains limited. In the pivotal adolescent semaglutide trial, semaglutide 2.4 mg weekly was generally well tolerated, although gastrointestinal adverse events were common and gallbladder-related events were reported (1). The current U.S. prescribing information also identifies acute pancreatitis and acute gallbladder disease as important safety considerations and reports a higher incidence of cholelithiasis and cholecystitis among pediatric patients aged 12 years and older receiving semaglutide compared with adults (3).

Adult evidence provides additional context. In the STEP 1 trial of semaglutide 2.4 mg weekly in adults with overweight or obesity, acute pancreatitis was reported in a small number of participants, while gallbladder-related events were also observed (6). In addition, post-marketing pharmacovigilance data have identified reports of pancreatitis associated with glucagon-like peptide-1 receptor agonists, although spontaneous reporting data cannot establish causality because of reporting bias, confounding, and the absence of a denominator (7).

Individual case reports have also described acute pancreatitis during semaglutide therapy. Hughes et al. reported acute pancreatitis considered likely related to semaglutide in an adult patient without an identifiable alternative cause, with improvement following discontinuation (8). Another case report described acute pancreatitis after transition from semaglutide to tirzepatide, further illustrating the clinical concern regarding pancreatitis during incretin-based therapy, although the specific causality and mechanism remain uncertain (9). These adult reports differ from the present case because our patient had documented gallstones before the episode and subsequently underwent cholecystectomy, making biliary pancreatitis the most clinically plausible etiology.

To our knowledge, reports specifically describing acute biliary pancreatitis occurring during semaglutide therapy in an adolescent with documented cholelithiasis remain limited. The present case therefore adds to the literature by illustrating the clinical complexity of attributing pancreatitis to semaglutide when established biliary risk factors coexist. In adolescents receiving glucagon-like peptide-1 receptor agonists, particularly those experiencing substantial weight loss or those with known gallstones, clinicians should maintain awareness of symptoms suggestive of biliary disease and pancreatitis. Further pediatric studies are needed to better characterize the incidence, risk factors, and mechanisms underlying biliary complications during glucagon-like peptide-1 receptor agonist therapy.

## Conclusion

This case highlights the clinical complexity of acute biliary pancreatitis occurring during semaglutide therapy in an adolescent with documented cholelithiasis. Although a definitive causal relationship between semaglutide and pancreatitis cannot be established, the temporal relationship following dose escalation, together with substantial weight loss during glucagon-like peptide-1 receptor agonist therapy, raises the possibility of a contributory role. Clinicians should remain vigilant for symptoms of gallbladder disease and pancreatitis in adolescents receiving glucagon-like peptide-1 receptor agonists, particularly those with known gallstones or substantial weight loss. Further studies are needed to better characterize the incidence and risk factors for biliary complications in this population.

## Data Availability

The raw data supporting the conclusions of this article will be made available by the authors, without undue reservation.
